# How sex and age affect immune responses, susceptibility to infections, and response to vaccination

**DOI:** 10.1111/acel.12326

**Published:** 2015-02-26

**Authors:** Carmen Giefing-Kröll, Peter Berger, Günter Lepperdinger, Beatrix Grubeck-Loebenstein

**Affiliations:** Institute for Biomedical Aging Research of Innsbruck UniversityInnsbruck, Austria

**Keywords:** hormone replacement, immunosenescence, infectious diseases, menopause, sex differences, vaccination

## Abstract

Do men die young and sick, or do women live long and healthy? By trying to explain the sexual dimorphism in life expectancy, both biological and environmental aspects are presently being addressed. Besides age-related changes, both the immune and the endocrine system exhibit significant sex-specific differences. This review deals with the aging immune system and its interplay with sex steroid hormones. Together, they impact on the etiopathology of many infectious diseases, which are still the major causes of morbidity and mortality in people at old age. Among men, susceptibilities toward many infectious diseases and the corresponding mortality rates are higher. Responses to various types of vaccination are often higher among women thereby also mounting stronger humoral responses. Women appear immune-privileged. The major sex steroid hormones exhibit opposing effects on cells of both the adaptive and the innate immune system: estradiol being mainly enhancing, testosterone by and large suppressive. However, levels of sex hormones change with age. At menopause transition, dropping estradiol potentially enhances immunosenescence effects posing postmenopausal women at additional, yet specific risks. Conclusively during aging, interventions, which distinctively consider the changing level of individual hormones, shall provide potent options in maintaining optimal immune functions.

## Introduction

A paradoxical situation between aging male and female is evident in particular regarding health status and the corresponding survival rates (Oksuzyan *et al*., 2008). Women exhibit significantly higher life expectancies, while frequently experiencing disabilities earlier in life (Van Oyen *et al*., 2013). Independent of age, death rates due to cancer, heart disease, accidents, influenza, or pneumonia are significantly higher in men (Austad, 2006). Here, major contributors appear to be lifestyle differences: smoking accounts for ∼60% of gender-specific mortality in Europe, alcohol abuse for ∼30% (McCartney *et al*., 2011), followed by risk-taking and seeking and complying with medical therapies (Oksuzyan *et al*., 2008). Besides that, major biological determinants of the sexual dimorphism in longevity are X chromosome diploidy, as well as distinct differences in immune and hormone responses (Vina *et al*., 2005; Austad, 2006; Oksuzyan *et al*., 2008), the latter both showing consistent changes with aging.

Women carry two X chromosomes. The X chromosome expresses several genes implicated in immunological processes, such as Toll-like receptors, multiple cytokine receptors, genes involved in T-cell and B-cell activity, and transcriptional and translational regulatory factors (Fish, 2008), while in turn the Y chromosome encodes for a number of inflammatory pathway genes, which are exclusively expressed in men (Flanagan, 2014). Most alleles on one X chromosome are randomly silenced during X chromosome inactivation already during embryogenesis. Polymorphism of X-linked genes and cellular mosaicism for X-linked parental alleles may offer additional advantages to women during host responses, in particular by providing a more adaptive and balanced cellular machinery during innate immune responses (Spolarics, 2007; Libert *et al*., 2010). Here, we therefore reviewed the current consistent knowledge on aging and sex-dimorphic susceptibility to infectious diseases and vaccination response in the context of changing levels of sex steroid hormones.

## Sexual dimorphic susceptibility to infectious diseases

Sex-specific infection and mortality rates have been documented in humans (Table1). This sexual dimorphism commences already during intrauterine development, for example, a male fetus experiencing a chronic inflammatory environment primarily being induced by the maternal immune system in the male placenta via decidual sites yet also likely due to a higher gestational infection rate of male placenta [(Goldenberg *et al*., 2006), reviewed in detail in (Clifton, 2010)]. Later in life very probably due to socioeconomic behavior, such as higher pathogen exposure during agricultural or occupational activities, men are more susceptible to many infections caused by viruses, bacteria, parasites, and fungi. They are significantly more predisposed especially to environmental and vector-borne diseases such as leptospirosis (3.5- to 4-fold increased incidence), schistosomiasis (1.5-fold), brucellosis, or rabies (Corbel, 2006; Guerra-Silveira & Abad-Franch, 2013). Albeit at comparable exposure levels, men exhibit higher incidence rates for leishmaniasis (2.5- to 3.5-fold increased incidence), pulmonary tuberculosis (2- to 3-fold), and hepatitis A (1.4-fold), again surmising the role of distinct biological differences (Guerra-Silveira & Abad-Franch, 2013). Similarly, premenopausal women succumb less often to meningococcal or pneumococcal infections. Testosterone exerts immune suppressive effects thereby increasing the severity of malaria, leishmaniasis, amebiasis, and tuberculosis, while at the same time supporting the clearance of toxoplasmosis (Bernin & Lotter, 2014; Nhamoyebonde & Leslie, 2014).

**Table 1:**
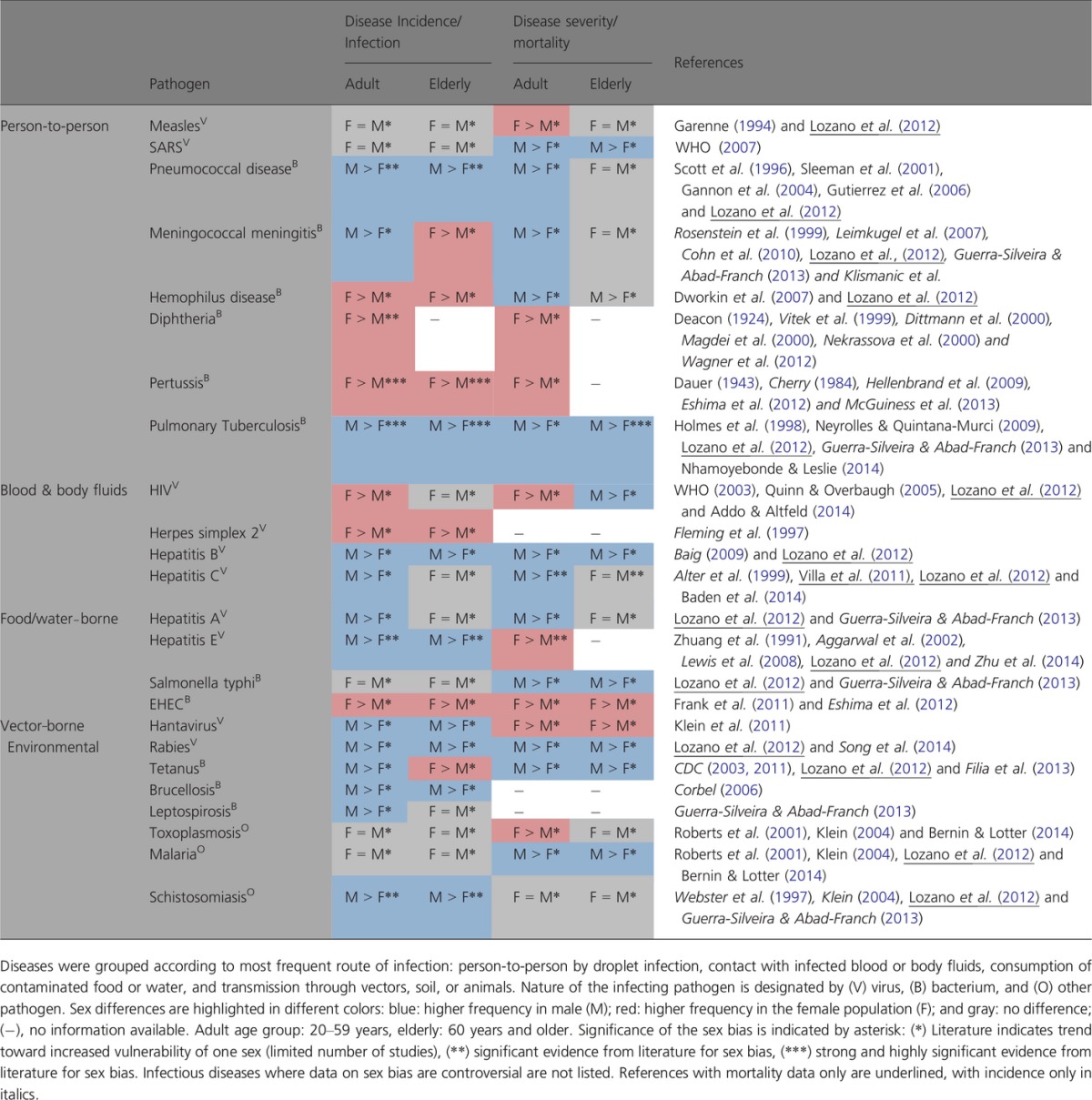
Sex specificity in infectious disease incidence and mortality

In contrast, sexually transmitted diseases occur more frequently and severely in women during their reproductive years supposedly due to behavior, sex-related mechanisms in reproduction and sex-specific steroid hormone levels (Rakasz & Lynch, 2002). The susceptibility to certain genital tract infections is further increased when applying oral contraceptives (Mohllajee *et al*., 2006). Once infected with HIV, progression toward AIDS was found to be faster in women (Meier *et al*., 2009; Addo & Altfeld, 2014). Variant incidence rates and severity regarding herpes zoster in women remain controversial (Thomas & Hall, 2004; Yawn & Gilden, 2013). Influenza shows a sex-biased incidence, yet besides elusive countrywise discrepancies, also strainwise differences remain to be resolved. Notably, however, men seem to be more affected by seasonal influenza (Lozano *et al*., 2012; Quandelacy *et al*., 2014), whereas premenopausal women succumb more often to pandemic strains (Serfung *et al*., 1967; Klein *et al*., 2010b,c; Klein *et al*., 2012). In cases of measles, toxoplasmosis, dengue, or hantavirus infections, incidence rates are not sex-biased. However, disease outcome was significantly worse in women, especially during reproductive years suggesting a decisive role for gonadal sex hormones (Garenne, 1994; Roberts *et al*., 2001; Klein *et al*., 2011; Guerra-Silveira & Abad-Franch, 2013). In women, elevated induction of pro-inflammatory cytokines and chemokines correlated with higher morbidity and mortality due to influenza infections (Klein *et al*., 2012). Again in women with ‘Escherichia-coli-O104:H4-associated-hemolytic-uremic-syndrome’, the humoral response was found stronger leading to detrimental effects most likely due to the overwhelming production of pathogenic antibodies (Frank *et al*., 2011; Greinacher *et al*., 2011).

It is well known that the aging process affects sexual dimorphism regarding immunocompetence and disease susceptibility. Aging women lose their immunological advantage. Particularly, they show increased susceptibility and mortality with respect to hepatitis, meningococcal, or pneumococcal infections (Table1). Protective effects of estrogen are thought to enable premenopausal women to clear the hepatitis C virus and thus progress slower to the disease than age-matched men. After menopause, these sex differences were lost and treatment efficacy was severely decreased (Khattab & Eslam, 2012; Baden *et al*., 2014). Notably, however, immune-pathological effects may also decrease after menopause, for example, in severe forms of dengue and influenza (Klein *et al*., 2012; Guerra-Silveira & Abad-Franch, 2013).

## Sex steroid hormones modulate immunocompetence

The immune system comprises innate and adaptive defense strategies. Innate immunity is the first line of defense and recognizes microbe-specific structures triggering phagocytosis, cell lysis, and secretion of cytokines. The latter stimulates an adaptive cellular immune response, which by means of cytokine secretion and antibody production specifically eradicates pathogens and infected cells.

Already in 1898, a potential link between sex hormones and the immune system has been proposed by Calzoari. He recognized changes in the thymus after castration (Calzoari, 1898). More observations along these lines were described later on: women have more CD4^+^ T cells (Amadori *et al*., 1995) and higher levels of circulating immunoglobulins (Ig), in particular IgM (Butterworth *et al*., 1967; Rhodes *et al*., 1969; Grundbacher, 1972). Also, women suffer more often from autoimmune diseases (Jacobson *et al*., 1997; Whitacre *et al*., 1999). Sex steroid hormones are thought to contribute to differences in humoral and cellular responses to infection and vaccination in men and women (Cook, 2008).

**Fig 1:**
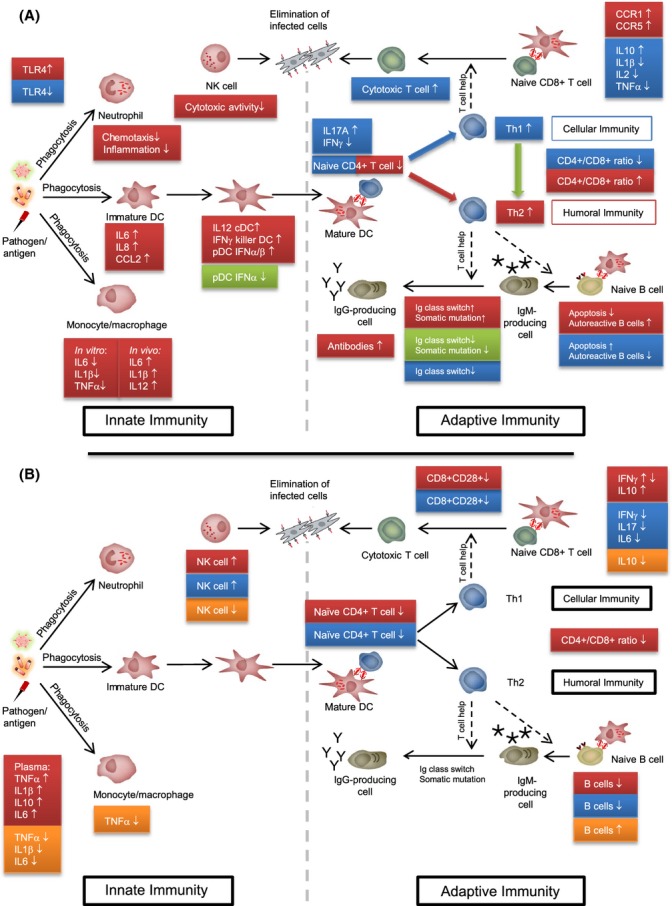
(A) Effects of sex steroid hormones on the immune system. Estrogens, in particular estradiol, and androgens, in particular testosterone, and progesterone affect cells of the innate and adaptive immune system. Effects of estrogens at ovulatory to pregnancy levels (during the reproductive phase of life) are shown in red, effects of testosterone in blue, and effects of progesterone in green. (B) Sex and aging affect the immune system. Sex-specific differences are resulting from genetic differences and changing sex steroid hormone levels especially during the menopause transition. Hormone replacement therapy (HRT) in women could partially revert these age-related changes of the immune system. Age-related changes in elderly women (postmenopause) are shown in red, in elderly men in blue, and effects of HRT on the female immune system in orange. Arrows indicate if cytokine levels, cell numbers, receptor expression, or biological functions are up (↑)- or down (↓)-regulated by the action of sex steroid hormones. DC, dendritic cell; NK, natural killer cell.

Gonadal hormones exert specific effects on the male and female immunocompetence at both the cellular and the molecular level (Fig.1A). Estrogen receptors are expressed in most cells of the innate and adaptive immune system including T cells, B cells, neutrophils, macrophages, dendritic cells (DC), and natural killer (NK) cells (Fish, 2008). Androgen receptors were identified in T and B lymphocytes. During pregnancy, activated lymphocytes also express progesterone receptors (Bouman *et al*., 2005).

Estrogens affect innate immune cells. Estrogens at levels of ovulatory phase or pregnancy suppress cytotoxicity of NK cells (Hao *et al*., 2007). Notably, macrophages treated *in vitro* with estradiol showed decreased secretion of the proinflammatory cytokines interleukin (IL)-1β, IL-6, and tumor necrosis factor (TNF)-α (Kramer *et al*., 2004), whereas long-term *in vivo* administration led to increased secretion of IL-1β, IL-6, and IL-12p40 after Toll-like receptor (TLR) 4 activation and eventually an enhanced activation status (Calippe *et al*., 2008). *In vitro* monocytes produce more IL-1β, IL-12, and TNF-α (Bouman *et al*., 2005; Gonzalez *et al*., 2010). Estradiol increases the anti-inflammatory and decreases the chemotactic activity of neutrophils (Ashcroft *et al*., 1999). Estrogen receptor signaling also regulates lineage development of DCs (Seillet *et al*., 2013). High estradiol levels promote the development of conventional IL-12-producing DCs and the expansion of IFN-γ-producing killer DCs. In addition, production of IL-6, IL-8, and chemokine (C-C motif) ligand 2 (CCL2) by immature DCs is increased (reviewed in Fish, 2008). In plasmacytoid DCs, the production of type 1 interferons (IFN) is enhanced in response to viral or exogenous nucleic acids via TLR7 and TLR9 (Seillet *et al*., 2012), whereas progesterone downregulates TLR9-mediated IFN-α production in response to viral infections potentially increasing a woman's risk for HIV and herpes simplex virus (HSV) (Hughes *et al*., 2008). In innate immune cells, testosterone decreases while estradiol increases the expression of TLR4 (Rettew *et al*., 2008, 2009).

Both estrogens and androgens reduce the numbers of immature T lymphocytes enhancing thymic involution during puberty and pregnancy (Tanriverdi *et al*., 2003; Olsen & Kovacs, 2011). Also adaptive immunity differs between men and women (Fig.1A): Androgens stimulate the development of Th1 responses and activate CD8^+^ T cells, whereas estrogens stimulate Th2 responses and activate antibody production (Giron-Gonzalez *et al*., 2000; Fish, 2008; Gonzalez *et al*., 2010). In women at reproductive age compared to age-matched men, the CD4^+^/CD8^+^ ratio is significantly increased (Seli & Arici, 2002). Low estrogen levels are paralleled by an increased expression of the transcription factor T-bet (T-box expressed in T cells), which in due course shifts the balance toward Th1 immunity and IFN-γ expression. High estrogen levels inhibit IRF1 (interferon regulatory factor 1) favoring Th2 immunity and IL-4 expression (Fish, 2008). This distinct shift from Th1 toward Th2 as well as the activity of regulatory T cells is enhanced by high progesterone levels during pregnancy, often leading to autoimmune disease remission yet also an elevated susceptibility toward certain infectious diseases, for example, influenza or malaria (Klein *et al*., 2010a; Pazos *et al*., 2012). Partially controversial results were reported regarding hormonal effects on the cytokine secretion pattern of T lymphocytes (reviewed in Bouman *et al*., 2005; Straub, 2007; Gonzalez *et al*., 2010; Oertelt-Prigione, 2012). Interestingly, no effects of sex steroid hormones on IL-4 and IL-10 production could be found so far. Data on the secretion of IFN-γ and IL-2 are conflicting (Bouman *et al*., 2005; Straub, 2007; Oertelt-Prigione, 2012). Naïve CD4^+^ T cells from women proliferated faster while producing more IFN-γ and less IL-17A compared to male T cells; this sex-specific cytokine imbalance was mediated by androgens (Zhang *et al*., 2012). Significantly, more regulatory T cells were found during the menstrual cycle when estrogen levels are high (Arruvito *et al*., 2007). Estrogen levels also play a role in T-cell homing, by enhancing the expression of chemokine receptors CCR1 and CCR5 in CD4^+^ cells (Yung *et al*., 1997; Mo *et al*., 2005). Testosterone increases IL-10 production, and men with androgen deficiencies have higher levels of IL-1β, IL-2, TNF-α, antibody titers, and CD4^+^/CD8^+^ T-cell ratios (Klein *et al*., 2010a).

Sex steroid hormones also modulate B-cell development and function (Fig.1A, reviewed in Sakiani *et al*., 2013). Estrogens and androgens suppress B lymphopoiesis in the bone marrow. Estradiol reduces apoptosis of immature B cells and thus increases the emergence of autoreactive B cells from central and peripheral checkpoints. However, estradiol also increases somatic hypermutation and class-switch recombination leading to high-affinity Ig-producing cells. These effects might contribute to an improved humoral response in women and explain the increased susceptibility to autoimmune diseases. In contrast to estrogens, progesterone suppresses somatic hypermutation and class-switch recombination (Sakiani *et al*., 2013). In the presence of androgens, apoptosis of immature B cells was enhanced and efficient class-switch recombination was inhibited, potentially limiting IgM to IgG conversion but also the pathogenicity of autoimmune reactions (Klein *et al*., 2010b; Sakiani *et al*., 2013). Estrogens also exhibit indirect effects on the immune system by modulating the levels of growth hormone, prolactin, or thymosin. Besides that, it is generally accepted that sex steroid hormones influence lifespan and healthy life expectancy also through other pathways (reviewed in Regan & Partridge, 2013).

Thus, the general paradigm on sex steroid hormones influencing the immune system stipulates that estrogens have immune-enhancing effects. In contrast, progesterone and androgens such as testosterone exert mainly immunosuppressive properties.

## Age and sex-specific changes of the immune system

The immune as well as the endocrine system experiences profound changes with aging, thereby increasing the susceptibility to infectious diseases (Gavazzi & Krause, 2002) and decreasing the efficacy of vaccination (Grubeck-Loebenstein *et al*., 1998). Immunosenescence impacts on the function of the innate as well as the adaptive immune system (reviewed in Panda *et al*., 2009; Ademokun *et al*., 2010; Agrawal & Gupta, 2011; Arnold *et al*., 2011; Mahbub *et al*., 2011; Weinberger & Grubeck-Loebenstein, 2012; Scholz *et al*., 2013). The functional capacity of innate immune cells declines. A diminished phagocytic capacity of DCs also leads to impaired antigen presentation and activation of the adaptive immune system. Involution of the thymus decreases the output of naïve T cells, and antigen-experienced T cells accumulate constricting the T-cell repertoire. Highly differentiated effector T cells produce more pro-inflammatory cytokines, which together with activated innate immune cells contribute to a systemic pro-inflammatory milieu in older age. Thus, developing specific vaccines and adjuvant immunization strategies appears imperative to ensure healthy aging (Boraschi *et al*., 2013).

Both men and women at advancing age exhibit reduced abilities to mount appropriate antibody responses especially toward new antigens. So far, sex-specific differences in the aging immune system and the effect of declining estrogen and progesterone levels on immunosenescence are poorly understood. At menopause, estradiol production in the ovaries ceases. Thereafter, only basal levels of progesterone are being synthetized by the adrenal glands. In aged women, dehydroepiandrosterone (DHEA) and testosterone levels decrease, yet follicle-stimulating hormone (FSH) and luteinizing hormone (LH) levels rise from the 4th decade onwards (Al-Azzawi & Palacios, 2009). In men, there is a slower yet steady decline in testosterone levels from their 2nd to 8th decade of life displaying no clear turning point (Bhasin *et al*., 2011). In turn, estradiol, estrone, LH, and FSH gradually increase (Morley *et al*., 1997; Jasuja *et al*., 2013).

The menopause has a distinct impact on the female immune system (Fig.1B). Postmenopausal women exhibit a reduced number of total lymphocytes, mainly B and CD4^+^ T lymphocytes (Giglio *et al*., 1994). Similarly, after surgical menopause, the CD4^+^/CD8^+^ ratio and the numbers of circulating B cells are decreasing, while NK cells are increasing (Kumru *et al*., 2004). Pronounced endocrine changes alter the expression of inflammatory mediators thereby elevating plasma IL-1β, IL-6, IL-10, and TNF-α with menopause (Deguchi *et al*., 2001; Kamada *et al*., 2001a; Vural *et al*., 2006; Yasui *et al*., 2007). After a transient rise in postmenopausal women, IFN-γ levels gradually decrease with age. Yet the production of IL-10 increases during the postmenopausal period (Deguchi *et al*., 2001).

Functional aspects of age/sex-specific differences of the immune system and its interplay with changing sex steroid hormone levels have not been investigated extensively (Fig.1B). Transcriptomic analysis of peripheral mononuclear blood cells of young and elderly individuals revealed that significantly more pathways were altered in women than men in particular highlighting lower T-cell defense and more inflammation in female seniors (Marttila *et al*., 2013). Women of reproductive age were shown to have significantly higher CD4^+^ T-cell and lower NK-cell counts, and at older ages, postmenopause reference ranges were comparable for both sexes (Jentsch-Ullrich *et al*., 2005). Yet another study showed age-related changes in lymphocyte subsets suggesting a faster progression of immunosenescence in men with a more pronounced decline of T cells including naïve CD4^+^ and CD8^+^CD28^+^, B cells, T-cell proliferative capacity, and IL-6 secretion, and a weaker increase in CD4^+^ memory T cells and NK cells (Hirokawa *et al*., 2013). Age-related alterations in T-cell cytokine secretion were also described to be sex specific, as IFN-γ and IL-17 secretion were decreased in elderly men after *in vitro* stimulation but not in women (Goetzl *et al*., 2010). IL-10 secretion was increased in elderly women but not in men (Pietschmann *et al*., 2003).

Hormone replacement therapy (HRT) in women showed beneficial effects on the immune system, as menopause-related immunological changes were partly reversed (Fig.1B). Estrogens especially increased B-lymphocyte numbers and decreased pro-inflammatory cytokine production (Kamada *et al*., 2000, 2001b; Porter *et al*., 2001). Baseline elevated IL-6 levels were significantly decreased, also was the production of IL-6 in stimulated PBMCs (Berg *et al*., 2002; Saucedo *et al*., 2002). Estradiol HRT reversed the postmenopausal increase in NK-cell activity (Albrecht *et al*., 1996) and reduced LPS-induced TNF-α production in monocytes (Aune *et al*., 1995). HRT also resulted in a significant reduction in plasma TNF-α and IL-1β levels (Vural *et al*., 2006) and IL-10 production (Deguchi *et al*., 2001). Yet the effect of estradiol on the increased secretion of Type 2 vs. Type 1 cytokines remains controversial (Kumru *et al*., 2008; Xia *et al*., 2009). Potential effects of testosterone replacement therapy on the immune system have not been addressed systematically. Its beneficial effects in elderly, in particular frail men, remain unclear as well as the risk of adverse events (Spitzer *et al*., 2013).

Age-related changes in sex steroid levels enhance immunosenescence-related alterations. In women, this can be partially reversed by HRT.

## Vaccine efficacy in men and women: is the sex bias maintained after menopause?

Sex-specific responses to distinct vaccines have been reported, unfortunately however solely in a minority of trials. Vaccination success in women, similar to stronger humoral and cellular responses after infection, is thought to be mediated through the action of sex steroid hormones (Cook, 2008). Based on 57 studies that actually stratified data according to sex, 54 of these studies reported clear vaccination side effects (Cook, 2009). Still large comprehensive data sets assessing sex-specific responses to vaccination are scarce, influenza vaccination being the sole exception. Before 1977, the FDA prohibited women in childbearing age to participate in phase 1 and 2 clinical trials, very probably this being still the reason why more or exclusively men are being enrolled. Since 1993, gendered analyses are required; however, reported data are sex-stratified only in rare cases (Simon, 2005; Geller *et al*., 2011).

In vaccinated adults, sex-related differences have been observed in immunogenicity and clinical effectiveness for influenza (Wang *et al*., 2002; Cook *et al*., 2006; Hui *et al*., 2006; Couch *et al*., 2007; Nichol *et al*., 2007; Engler *et al*., 2008; Falsey *et al*., 2009; Klein *et al*., 2010c, 2012; Talaat *et al*., 2010; Khurana *et al*., 2012; Furman *et al*., 2014), pneumococcal polysaccharide (Sankilampi *et al*., 1996; Wagner *et al*., 2003; Brandao *et al*., 2004; Cook *et al*., 2007; Goldblatt *et al*., 2009; Soneji & Metlay, 2011; Wiemken *et al*., 2014), hepatitis A and B (reviewed in Cook, 2008; Klein *et al*., 2010a), tetanus (Marvell & Parish, 1940), diphtheria (Hasselhorn *et al*., 1997), measles (Green *et al*., 1994), meningococcal (Edwards *et al*., 2008; Krasnicka, 2010), yellow fever (Monath *et al*., 2002; Pfister *et al*., 2005; Veit *et al*., 2009), rabies (Briggs *et al*., 2000; Banga *et al*., 2014), smallpox (Kennedy *et al*., 2009), Venezuelan equine encephalitis (Pittman *et al*., 1996), brucella (Rhodes *et al*., 1969), and HSV2 vaccine (Corey *et al*., 1999; Stanberry *et al*., 2002). Yet distinct documentation on sex-specific responses in probands of advanced age is extremely scarce. Those studies which have disclosed information on, or, specifically tackled coverage, adverse events, immunogenicity, and effectiveness are listed in Table2.

**Table 2:**
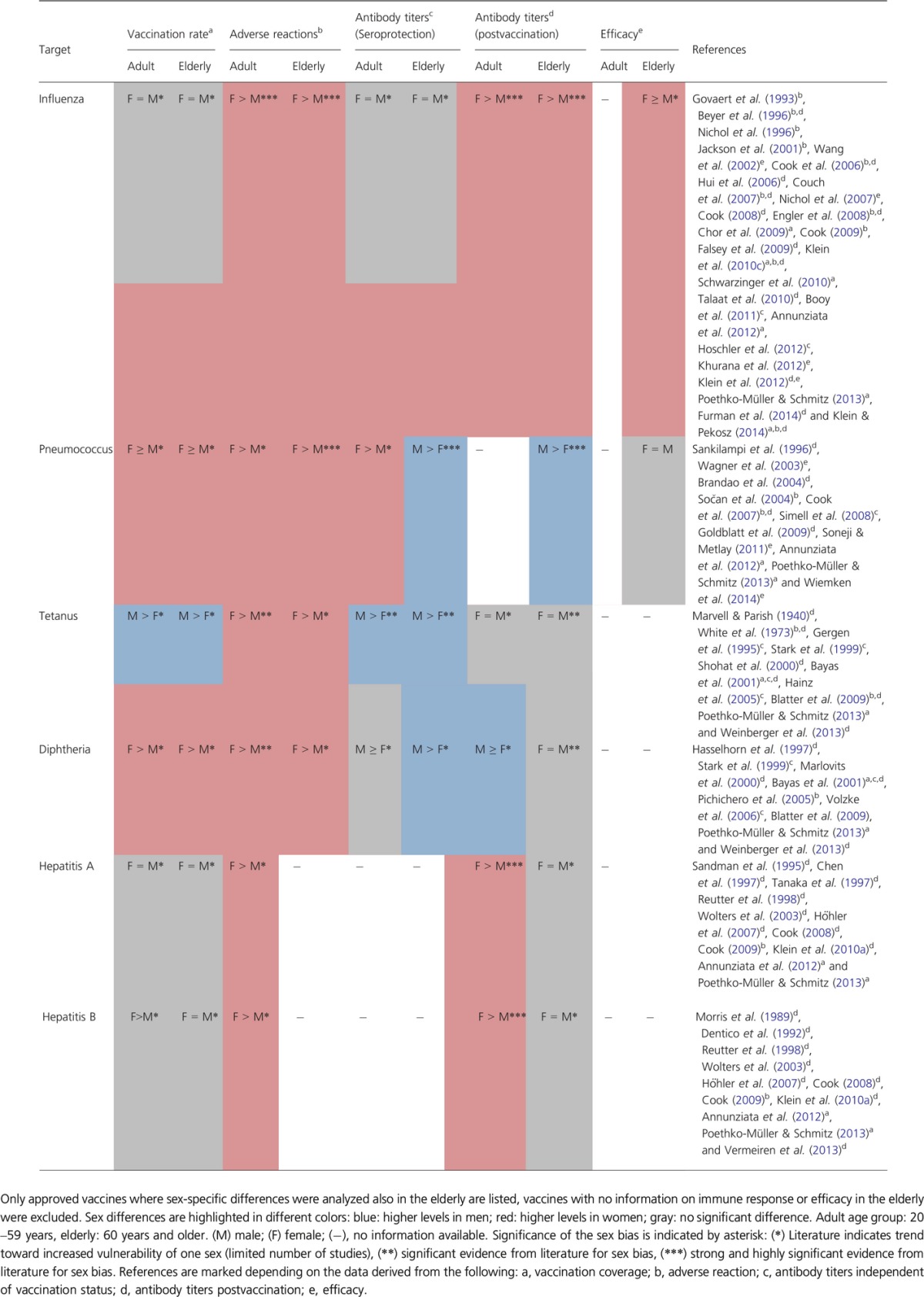
Sex and gender-related differences in vaccination

Despite higher morbidity and mortality during influenza pandemics, women actually show a better response to influenza vaccination, in particular by mounting higher levels of neutralizing antibodies (Wang *et al*., 2002; Cook *et al*., 2006; Nichol *et al*., 2007; Engler *et al*., 2008; Falsey *et al*., 2009; Klein *et al*., 2012; Furman *et al*., 2014). However, side effects were significantly more frequent in female vaccinees (Cook *et al*., 2006; Engler *et al*., 2008; Cook, 2009; Klein *et al*., 2012) perhaps due to a concomitant high expression of inflammatory cytokines (Furman *et al*., 2014). Women being vaccinated with half doses had geometric mean titers comparable to men having received a full dose (Engler *et al*., 2008), and men with high testosterone levels mounted lower titers of neutralizing antibody suggesting a immunosuppressive role for testosterone in the context of influenza vaccination (Furman *et al*., 2014).

Women are more susceptible to HSV2 (Table1). A recombinant glycoprotein D vaccine yielded a 38–42% efficacy in HSV1 seronegative women, yet was not efficacious at all in men and in HSV1 seropositive women (Stanberry *et al*., 2002). Studies in mice showed that estradiol increases the efficacy of HSV2 vaccine (Pennock *et al*., 2009). Women also react stronger to hepatitis B vaccination as 5% men after age 29 were nonresponders, whereas women only dropped to this level after age 43, yet exhibiting no actual advantage after menopause (Vermeiren *et al*., 2013).

Women are generally immune-privileged. Yet in some exceptions, men are better protected, for example, against diphtheria and tetanus (Table1). This correlates with higher levels of seroprotection (Table2). In this context, military immunizations and higher rates of injury with consecutive immunization in men are considered decisive (Gergen *et al*., 1995; Volzke *et al*., 2006). Protective antitetanus titers were found in 60% of men aged 70+ with a history of military service compared to 20–30% of not serving men and < 20% in women (Gergen *et al*., 1995). Diphtheria vaccination failed twice as often in women and resulted in significantly lower antibody titers (Hasselhorn *et al*., 1997). Yet prior to the introduction of common diphtheria vaccination, more boys than girls suffered from diphtheria, which interestingly reversed after puberty (Deacon, 1924).

In this context, also the type of antigen used for vaccination appears crucial. Naturally acquired immunity primarily provided by antipneumococcus protein-specific antibodies knows no sex difference during ages below 65. Interestingly, in women below 65, the titers of polysaccharide-specific antibodies were significantly higher (*P* < 0.05). Later in life this difference vanishes, and now, aged men had statistically significantly higher levels against the major surface proteins PspA and CbpA (*P* < 0.05) (Simell *et al*., 2008). Notably, vaccination with a pneumococcal polysaccharide vaccine (PPV23) mounted significantly (up to 30%) higher antibody levels in men of advanced ages (Sankilampi *et al*., 1996; Brandao *et al*., 2004; Goldblatt *et al*., 2009).

DHEA failed as a vaccination adjuvant in human trials (reviewed in Hazeldine *et al*., 2010). Despite that, HRT based on estrogens may increase responsiveness in elderly women as vaccination studies performed during different phases of the ovarian cycle in younger individuals showed significantly higher efficacy in line with high estrogen levels. Cholera vaccination during the follicular phase of the menstrual cycle was superior (Kozlowski *et al*., 2002). Also data from animal studies support the assumption that HRT may improve vaccine effectiveness. Vaccination of ovariectomized rhesus macaques was characterized by an increased frequency of terminally differentiated CD4^+^ effector memory T cells and inflammatory cytokine-secreting memory T cells as well as a reduction in T-cell cytokine and IgG production. This could be partly reversed by HRT (Engelmann *et al*., 2011). Estradiol also improved the efficacy of a vaccine against genital herpes in mice (Bhavanam *et al*., 2008; Pennock *et al*., 2009). The combination of estradiol and influenza vaccination induced neutralizing antibody titers in ovariectomized mice comparable to intact mice (Nguyen *et al*., 2011).

## Conclusion and future perspectives

Specific immune responses differ in aging men and women (Table3). Besides genetic factors, these differences can be explained through the greatly divergent and changing levels of sex steroid hormones and their interplay with the immune system. Estrogens promote while androgens suppress immune responses during infections, after vaccination or in case of autoimmunity. On these grounds, men are more susceptible against many taxa, while female suffer more from diseases with enhanced immunopathological impact. Studies on how aging affects these sex-related differences in immunity are rare. Based on epidemiological data, women appear to lose their immunological advantage after menopause. To what degree hormonal levels are effective in this context needs to be assessed in more detail. Data from animal studies suggest effects of HRT on vaccine efficacy, again requiring more careful evaluations in humans. Women at high estrogen levels commonly mount a stronger humoral response. This effect could however not be generalized for any vaccine type. Therefore, further improvement of vaccination response may be feasible in postmenopausal women as well as aging men. To date, most vaccines have been tested and adjusted in male probands. Hence, efforts in individualizing dosing and vaccination schedules may achieve enhanced protection and reduction of side effects in women. Although current clinical trials are sex-matched, sex-specific evaluations are rarely disclosed despite being highly desirable.

**Table 3:** Summary on how sex and age affect immune responses, susceptibility to infections, and response to vaccination

**Sex/Gender bias in infectious disease susceptibility:** Many infectious diseases: increased infection/mortality rates in men vs. women Few exceptions, for example, sexually transmitted diseases Some infectious diseases same incidence but more severe in women, for example, measles, toxoplasmosis, dengue, hantavirus (immunopathology) Aging partially alters sex bias →contribution of hormones	**Sex steroid hormones modulate the immune system:** Estrogens largely immunoenhancing effects Androgens and progesterone mainly immunosuppressive effects Effects of gonadal steroid hormones documented for adaptive (CD4^+^, B cells) and innate cells (NK, macrophages, DC) Cytokine secretion is affected Gonadal hormones influence the Th balance Estrogens enhance the production of high-affinity Ig
**Sexual dimorphism in immunosenescence:** Immune and endocrine system change with age Aging of the immune system differs in men and women Menopause in particular has a strong impact on the female immune system HRT partially reverses immune aging effects back to premenopausal levels confirming the influence of hormones	**Sex/Gender bias in vaccination:** Immune responses to some vaccines differ between men and women Often stronger humoral responses in women, for example, influenza, hepatitis B But also stronger responses in men, for example, pneumococcal polysaccharide vaccine Aging partially alters sex bias →contribution of hormones Animal models: HRT reverses vaccine efficacy back to premenopausal levels

In summary, the gender gap in life and healthy life expectancy stems from gender and sex-related differences. Both have to be addressed to achieve a longer and healthier life in humans: (i) behavioral factors related to rate of infection need to be altered thereby increasing vaccination coverage and booster immunizations, (ii) composition and dosage of vaccines need to be adjusted to comply with sex-specific differences in immunity, and (iii) preclinical data warrant human trials on combining short-term HRT with vaccinations to improve vaccine efficacy.

## Search strategy and selection criteria

The data presented in this review were compiled by searches of PubMed, the clinicaltrial.gov registry, Cochrane, FDA, and EMEA databases from November 2013 to September 2014 using the search terms (alone or in combination) ‘menopause’, ‘immunosenescence’, ‘sex or gender’, ‘immune system’, ‘estrogen or estradiol’, ‘testosterone’, ‘androgen’, ‘progesterone’, ‘sex hormone’, ‘women’, ‘hormone replacement therapy’, ‘vaccine or vaccination’, ‘sex difference’, ‘influenza’, ‘pneumococcus’, ‘herpes zoster or varicella’, ‘hepatitis’, ‘herpes simplex’, ‘tetanus’, ‘diphtheria’, ‘pertussis’, ‘encephalitis’, ‘vaccination rate or coverage’, ‘adverse reactions’, ‘seroprevalence or seroprotection’, ‘antibody or humoral’, and ‘efficacy or effectiveness’. Studies that failed to analyze differences by sex were excluded. Only papers published in English or German were used. Also, the primary literature of cited review articles was reevaluated.

## Funding

This work was supported by the European Union's Seventh Framework Program MOPACT (‘Mobilizing the potential of active aging in Europe’; FP7-SSH-2012-1 grant agreement no. 320333).

## Conflict of interest

None declared.
